# Enhancing Laparoscopic Simulation Skills Through Nondominant Hand Training in Daily Activities

**DOI:** 10.7759/cureus.108025

**Published:** 2026-04-30

**Authors:** Rita K Bliesner, Autumn A Stevens, Lukyn Holling, James R Nolin

**Affiliations:** 1 Department of Research, Alabama College of Osteopathic Medicine, Dothan, USA; 2 Department of Simulation, Alabama College of Osteopathic Medicine, Dothan, USA

**Keywords:** laparoscopic simulators, medical education, motor dexterity, nondominant hand use, surgical training

## Abstract

Background

Being in the large majority, right-handed people are not required to use their nondominant hands throughout their daily lives, resulting in a loss of motor dexterity. As technology continues to advance, improving motor dexterity in nondominant hands has proven to be not only advantageous but imperative for surgeons. In this study, we assessed how increasing the use of participants’ nondominant hands impacted proficiency during laparoscopic simulator tasks.

Methods

A total of 18 preclinical medical students, divided into experimental and control groups, were timed twice on three laparoscopic simulator tasks. The experimental group spent three weeks between testing phases using their nondominant hand for daily activities and recording compliance through a daily survey.

Results

Compared to the control, the experimental group showed a significant improvement in proficiency for two of the tasks (p<0.05). No participants in this group successfully completed the task requiring the greatest motor dexterity during either testing phase. Additionally, all nine experimental participants (100%) reported feeling substantially more confident in their laparoscopic abilities during the post-test.

Conclusion

This study examined whether incorporating nondominant hand use into daily activities could improve laparoscopic task performance among preclinical osteopathic medical students. Results suggest that short-term, self-directed ambidexterity training may enhance basic laparoscopic skills and learner confidence, though gains in fine motor control were limited. This study can encourage physicians and residency programs to incorporate nondominant hand use into their curriculum and everyday life.

## Introduction

In a world largely designed to accommodate the 90% of individuals classified as right-hand dominant, use of the nondominant left hand has become unnecessary [1-3]. As a result, motor coordination and functional proficiency of the nondominant hand are often underdeveloped. Even in cases of upper extremity peripheral nerve injury affecting the dominant hand, individuals continue to favor its use over the nondominant hand when performing everyday tasks, highlighting a strong behavioral preference for dominance regardless of functional impairment [4]. This habitual neglect of the nondominant hand contributes to reduced motor dexterity, which is defined as the coordination and control of fine motor movements [5]. Interestingly, individuals who are left-hand dominant tend to exhibit less discrepancy in task performance between their dominant and nondominant hands [6]. This reduced variability suggests that left-handed individuals may engage their nondominant (right) hand more frequently or effectively during daily activities [7]. As a result, the performance gap between hands is significantly smaller in left-handed individuals compared to their right-handed counterparts [6]. Despite the general underuse of the nondominant hand in daily life, there are numerous professions and skill-based activities that require effective bimanual coordination to achieve success. Fields such as music, fine arts, and particularly minimally invasive surgery demand high levels of ambidextrous proficiency. In these contexts, poor nondominant hand coordination can become a limiting factor in overall performance. Developing interventions that promote motor control and dexterity in the nondominant hand may therefore provide significant benefits for individuals training in these fields, especially in surgical disciplines where bimanual skill is critical.

Laparoscopic surgery has become a cornerstone of modern surgical practice due to its advantages in reducing postoperative pain, minimizing hospital stays, and improving cosmetic outcomes compared to open procedures. However, the technical demands of laparoscopic surgery are significant, requiring the development of advanced psychomotor skills, spatial awareness, and bimanual dexterity. Unlike open surgery, where tactile feedback aids surgical performance, laparoscopic procedures rely on indirect visualization and counterintuitive hand movements, often performed through long instruments with limited degrees of freedom. The steep learning curve required for trainees to become proficient can result in decreased performance, increased frustration, and slower skill acquisition if they enter training without prior laparoscopic simulation experience. In particular, proficiency in using the nondominant hand, a task not typically emphasized in everyday life, can pose a significant barrier to skill acquisition during laparoscopic simulation training. As technology continues to advance, improving fine motor skills in nondominant hands has proven to be not only advantageous but imperative for surgeons [8]. Motor learning can be enhanced by incorporating tasks that promote neural plasticity and hand coordination outside the clinical environment [9]. Engaging the nondominant hand in routine tasks may provide a low-cost, accessible method to improve dexterity and coordination in a nonclinical context, potentially transferring those benefits to laparoscopic performance.

Despite the theoretical benefits, it remains unclear whether increased nondominant-hand use in unstructured, real-world daily activities can translate into measurable improvements in laparoscopic simulation performance. Previous studies on ambidexterity and motor training have primarily focused on structured, task-specific, or simulation-based interventions, with limited investigation of everyday behavioral modification as a training strategy [10,11]. This study aims to evaluate the effect of structured nondominant hand use in daily activities on laparoscopic simulation performance among novice trainees. The primary outcomes assessed are task completion time and procedural proficiency during standardized laparoscopic simulation tasks. We hypothesize that participants who engage in intentional nondominant hand training will demonstrate improved performance, reflected by decreased task time and increased proficiency scores, compared to their baseline performance. By examining changes following a defined period of nondominant hand use, this study seeks to determine whether this accessible behavioral intervention can serve as a practical, low-cost adjunct to early surgical training that could be implemented outside of simulation environments to support the development of bimanual dexterity.

## Materials and methods

This was a study conducted at the Alabama College of Osteopathic Medicine, Dothan, Alabama, United States. The study was approved by the Alabama College of Osteopathic Medicine Institutional Review Board (approval number: 24-09-10-001).

Study population

Participants were recruited from preclinical osteopathic medical student cohorts and volunteered their time. Inclusion criteria consisted of preclinical osteopathic medical students who were right-hand dominant, had no prior extensive laparoscopic training, and provided informed consent. Exclusion criteria included left-hand dominance, previous formal surgical or laparoscopic experience, upper extremity injury or neurologic conditions affecting motor function, or inability to comply with study procedures. Participants were randomly assigned to either the experimental or control group using a computer-generated randomization sequence and were assigned unique identification numbers. Group allocation was available to participants after assignment; however, investigators responsible for timing the laparoscopic simulation tasks were blinded to group allocation during pre- and post-testing sessions. Both groups participated in the pre-test timing and post-test re-timing.

Study process

**Figure 1:**
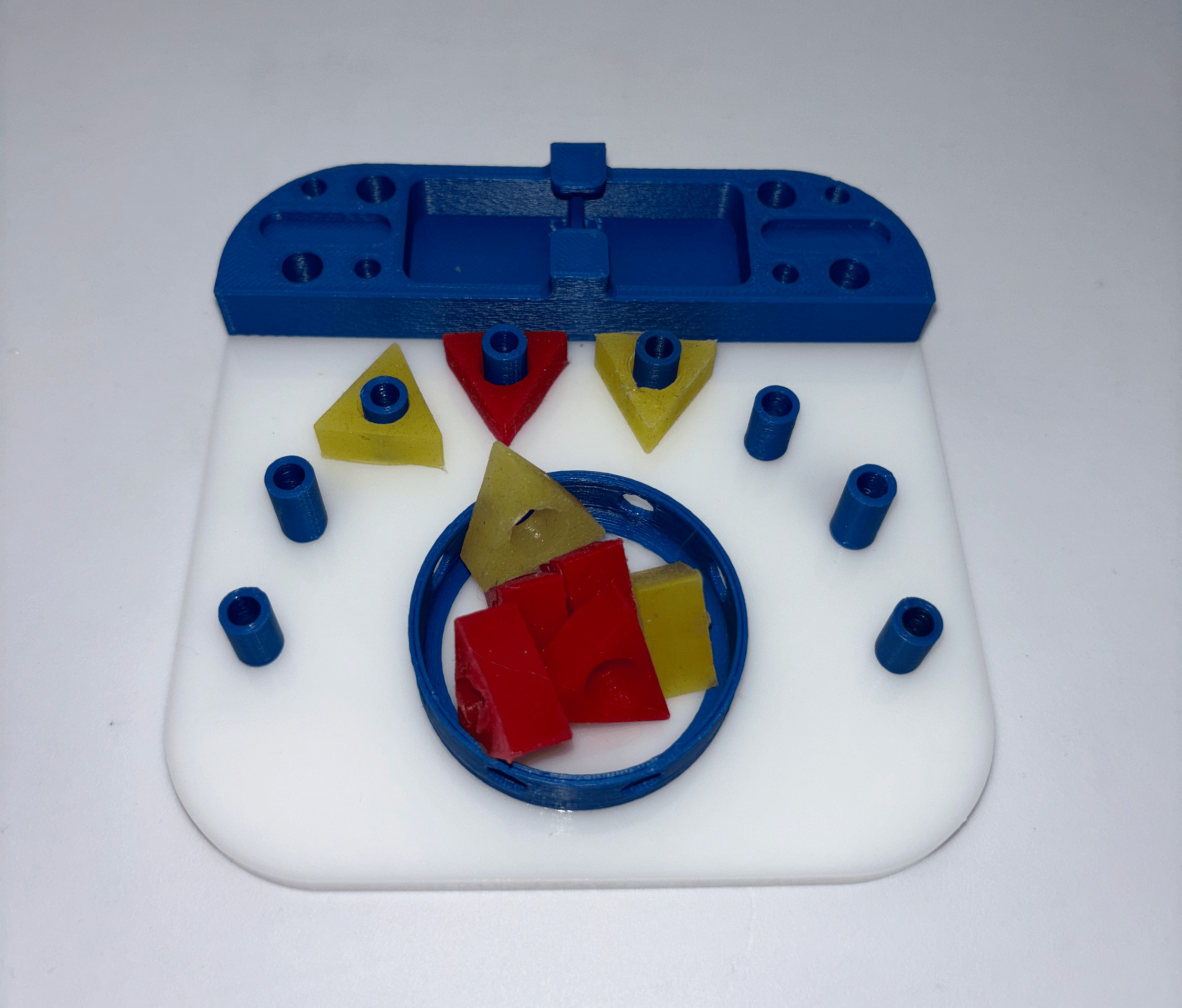
Triangle task setup. The triangle task required participants to grasp a triangle from the central well, transfer it between graspers, and place it onto a peg. The order of placement was left to the participant’s discretion. The color of the triangles was not relevant to the study. The triangles were made of a compressible plastic material, allowing for easy handling, stretching, and maneuverability. Photograph by author, 2025

**Figure 2:**
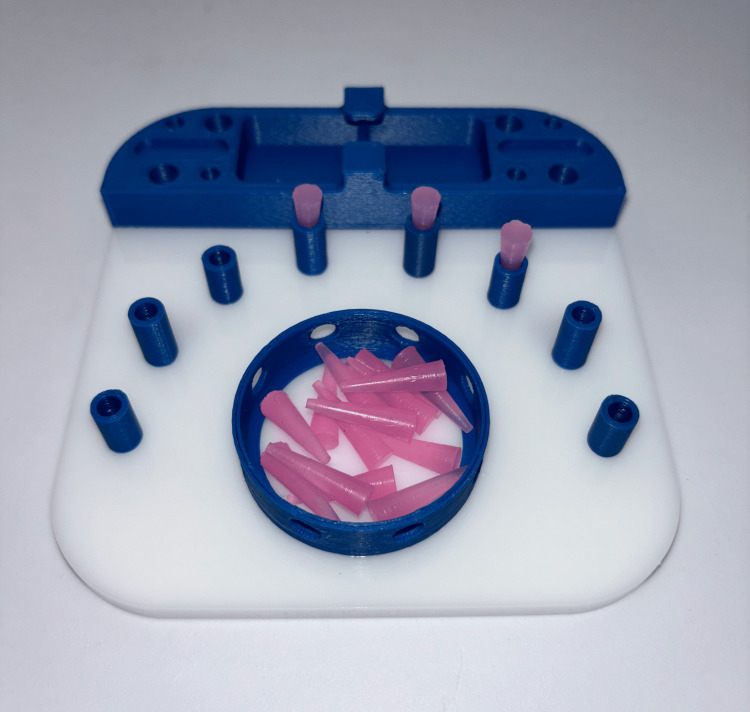
Cone task setup. The cone task required participants to grasp a cone from the central well, transfer it between graspers, and place it into a peg. The order of placement was left to the participant’s discretion. More cones than pegs were provided, so if a cone was dropped, it did not need to be retrieved to complete the task successfully. The cones were made of sturdy plastic with a slightly textured surface, allowing for relatively easy handling. Photograph by author, 2025

**Figure 3:**
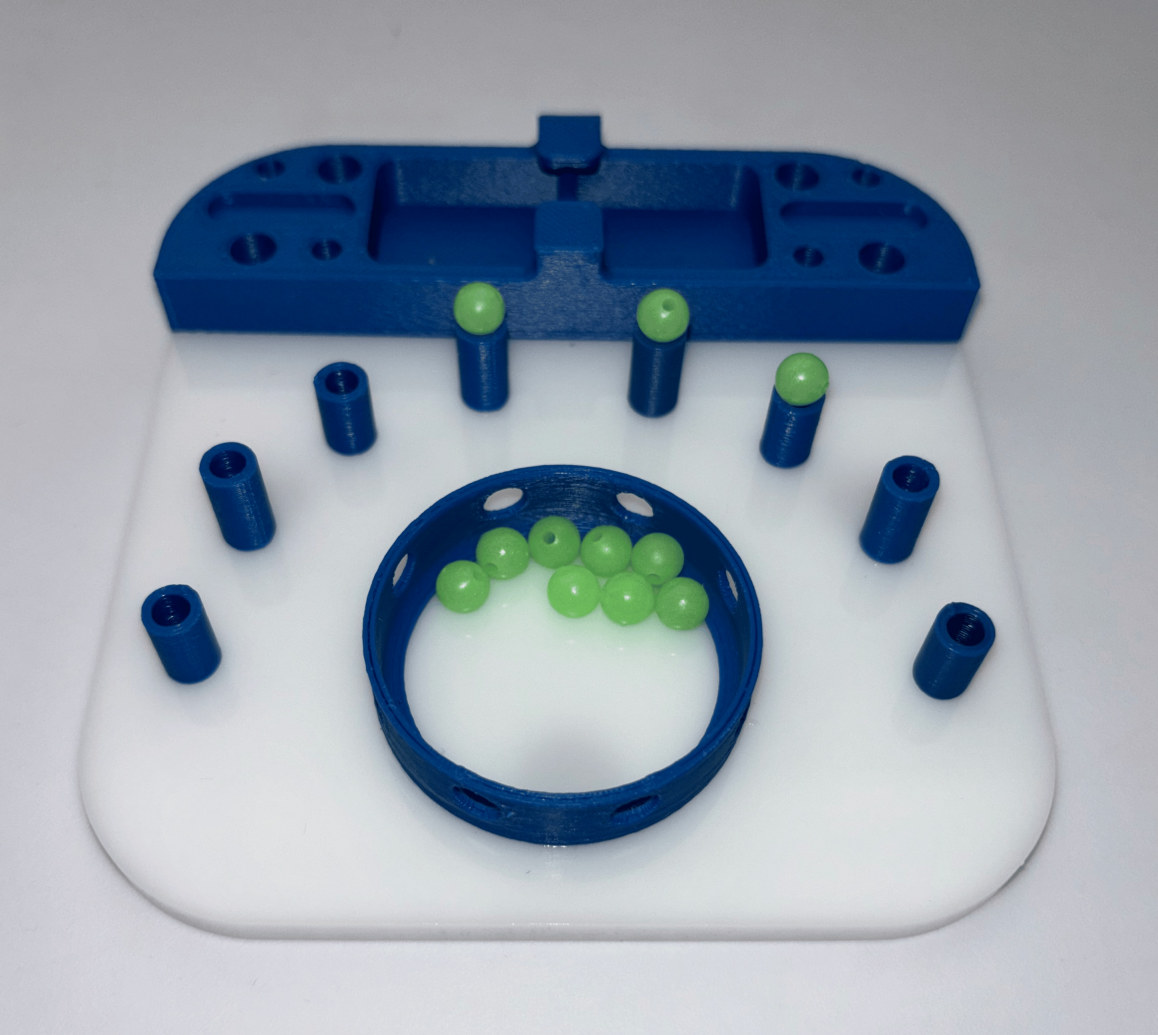
Ball task setup. The ball task required participants to grasp a ball from the central well, transfer it between graspers, and place it onto a peg. The order of peg placement was left to the participant’s discretion. As with the cone task, there were more balls than pegs, so dropping a ball did not necessitate retrieval to complete the task successfully. The balls were made of hard plastic, making them somewhat challenging to grasp. Photograph by author, 2025

Private rooms were set up with the Laparo Aspire laparoscopic simulator trainers (Laparo Sp. z o.o, Wrocław, Poland) on adjustable tables. Each participant was given an introduction to the machine and had five minutes to become familiar with the instruments and adjust the table height. They then completed three tasks in a randomized order. Each task required the participant to pick up an object with the fenestrated grasper, transfer it to the Maryland dissecting forceps, and then complete the task. The triangle task required the participant to take eight triangles from the center and place each triangle onto a peg (Figure 1). The cone task required the participant to pick up eight cones and insert each cone into the hole of a peg (Figure 2). The ball task required the participant to pick up eight balls and place each one on top of a peg (Figure 3). If the participant did not transfer the object successfully or dropped it, the object was to be placed back in the starting position, and they could continue their attempt. The time began as soon as the participant touched an object with the fenestrated grasper and ended when the task was completed. If the timer reached five minutes, the participant was asked to stop and move on to the next task, resulting in a “failure.” A “success” was characterized as moving all objects to their designated pegs within the allotted five minutes.

Data collection and measurements

Participants from the experimental group were instructed to use their nondominant hand for daily activities for three weeks to allow them enough time to become familiar with using their nondominant hand. A nightly survey was sent to participants in the experimental group to assess daily nondominant hand use. The survey was adapted from the Motor Activity Log [12] and used a 0-5 Likert-type scale with the following anchors: 0 = not used (did not use nondominant hand), 1 = occasionally used nondominant hand but very rarely, 2 = sometimes used nondominant hand but primarily used dominant hand, 3 = used both hands about equally, 4 = used nondominant hand more often than dominant hand, 5 = used nondominant hand exclusively. “N/A” was recorded when the activity was not performed that day. This survey was used to monitor self-reported compliance with the intervention. Compliance data were collected prospectively through nightly surveys but were not analyzed until after completion of the pre- and post-intervention performance assessments. Recommended activities included turning on a light switch, opening drawers, holding and scrolling on a phone, opening doors, unlocking doors with a key, using a remote, picking up a cup, brushing their teeth and hair, writing, using a fork or spoon to eat, and eating finger foods. Experimental participants were provided with pieces of string for them to tie to various items as reminders to use their nondominant hand. After the three-week experimental period, both groups were re-timed on the laparoscopic trainers using the same protocol for the post-test portion. All participants were sent a post-test survey to gather feedback on their experience and perceived improvement.

Statistical analysis

Statistical analysis included paired t-tests to assess within-group differences between pre-test and post-test performance and independent t-tests to compare differences between the experimental and control groups. A p-value of <0.05 was considered statistically significant.

## Results

A total of 16 out of the 18 (88.9%) participants identified as right-hand dominant. The other two participants classified themselves as ambidextrous; however, they primarily used their right hand for writing and throwing. A total of 17 of the 18 (94.4%) participants had never used a laparoscopic simulator trainer before. The remaining participant had only a brief introductory experience with the trainers from a prior event. Experimental participants showed an overall improvement in the total number of successes across all three tasks, increasing from 9/27 (33.3%) successfully completed in the pre-test to 15/27 in the post-test (55.6%) (Tables 1, 2). While the control group had more successes during the pre-test, the group saw a decrease in successful task completion between pre-test (16/27 successes; 59.3%) and post-test (13/27 successes; 48.1%) (Tables 1, 2). Three control group participants were able to complete the task with the balls, which requires the most motor dexterity, during the pre-test. However, none of the participants successfully completed that task within the five-minute window during the post-test re-timing. No experimental group participants successfully completed the ball task during either the pre- or post-test.

**Table 1:** Pre-test performance of the control and experimental groups Values are presented as n (% of group total)

Group	Triangles, n (%)	Cones, n (%)	Balls, n (%)	Total, n (%)
Control (n=9)	8 (88.9%)	5 (55.6%)	3 (33.3%)	16 (59.3%)
Experimental (n=9)	6 (66.7%)	3 (33.3%)	0 (0.0%)	9 (33.3%)
Total (n=18)	14 (77.8%)	8 (44.4%)	3 (16.7%)	

**Table 2:** Post-test performance of control and experimental groups Values are presented as n (% of group total)

Group	Triangles, n (%)	Cones, n (%)	Balls, n (%)	Total, n (%)
Control (n=9)	8 (88.9%)	5 (55.6%)	0 (0.0%)	13 (48.1%)
Experimental (n=9)	9 (100%)	6 (66.7%)	0 (0.0%)	15 (55.6%)
Total (n=18)	17 (94.4%)	11 (61.1%)	0 (0.0%)	

**Figure 4:**
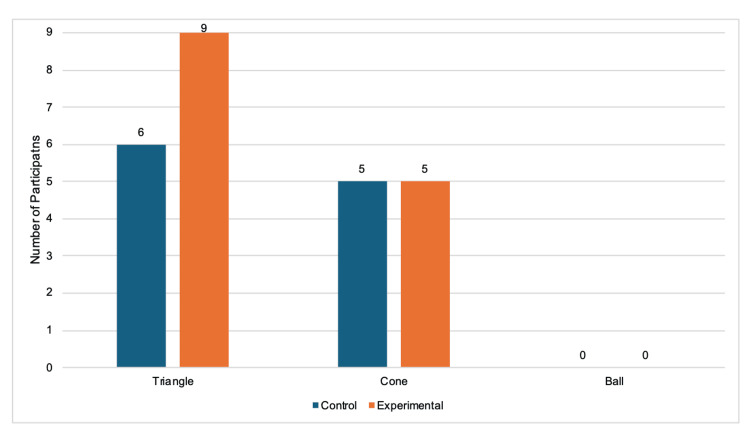
Number of participants who improved in post-test performance. Bars represent the number of individuals whose post-test response times were faster than their pre-test baseline. Only participants who improved are shown in the graph. Data are grouped by laparoscopic simulator task and colored by experimental condition.

**Figure 5:**
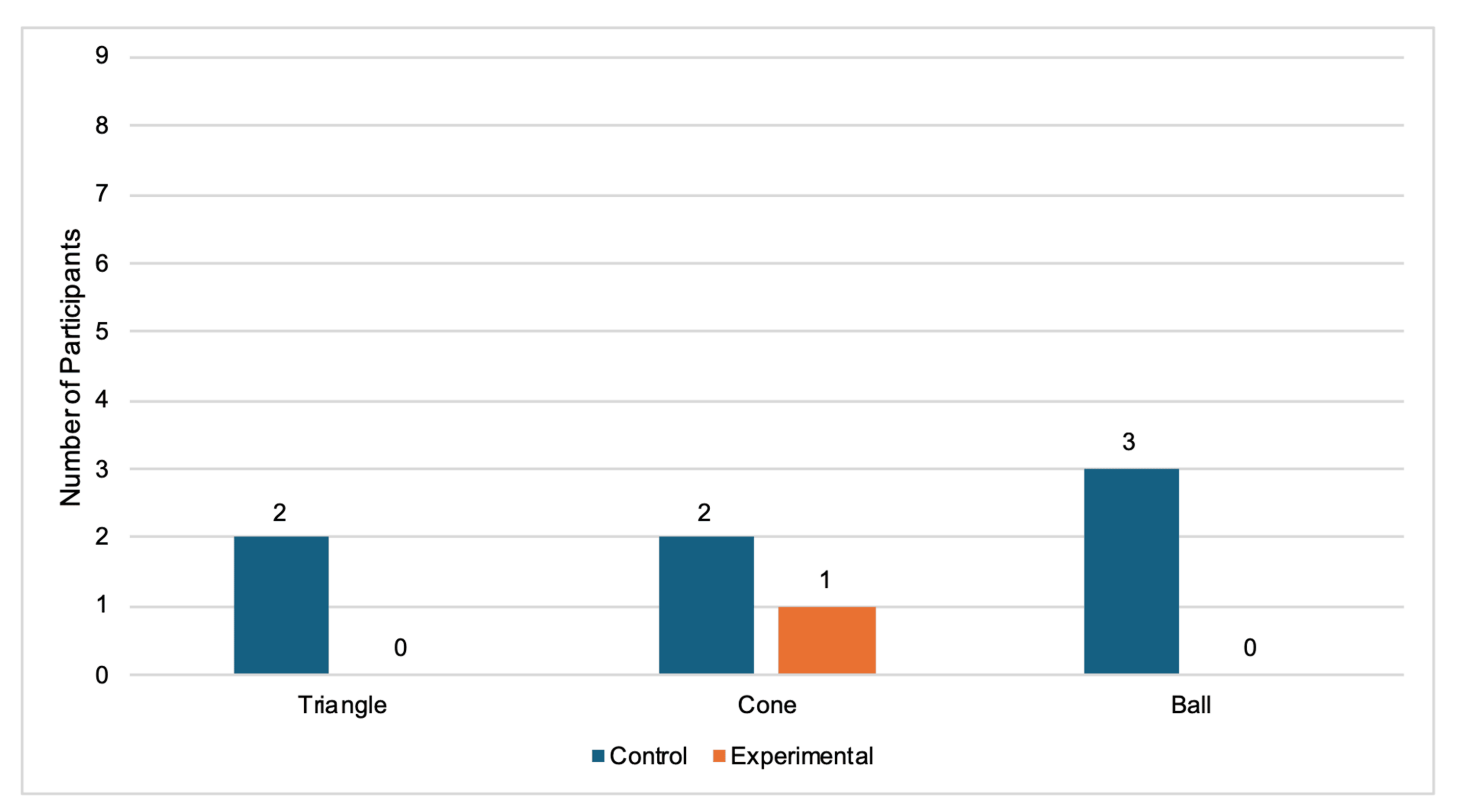
Number of participants who did not improve in post-test performance. Bars represent the number of individuals whose post-test response times were slower than their pre-test baseline. Only participants who did not improve are shown in the graph. Data are grouped by laparoscopic simulator task and colored by experimental condition.

**Figure 6:**
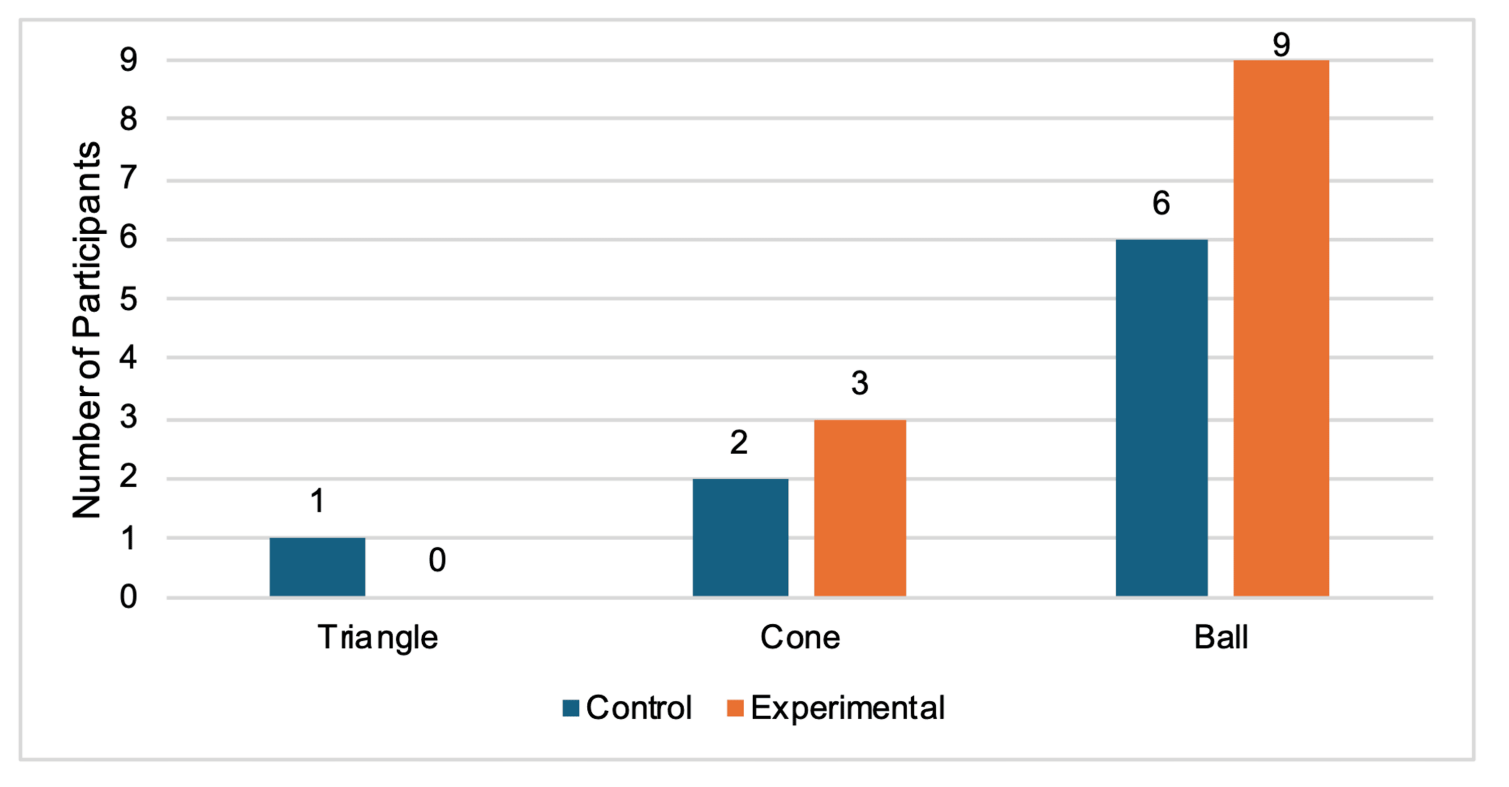
Number of participants who failed both the pre-test and post-test laparoscopic simulator trainer tasks. Bars represent the number of individuals who failed both test trials. Only participants who failed both are shown in the graph. Data are grouped by laparoscopic simulator task and colored by experimental condition.

When comparing individual performances between the pre-test and post-test, 9/9 experimental participants (100%) were faster at the triangle task, and 5/9 (55.6%) were faster at the cone task after the experimental period of using their nondominant hand for three weeks in daily activities (Figure 4). For the triangle task, 6/9 control participants (66.7%) were faster, and 5/9 (55.6%) were faster for the cone task during the second timing (Figure 4). No participants in either category got faster for the ball task (Figure 4). Only one of the nine (11.1%) experimental participants got slower between tests, specifically while completing the cone task. During both the triangle and cone tasks, 2/9 (22.2%) separate control participants had slower outcomes during the post-test (Figure 5). The 3/9 control participants (33.3%) who successfully completed the ball task during the pre-test were not able to finish within the five-minute timeframe and, therefore, were slower (Figure 5). From the control group, 1/9 (11.1%), 2/9 (22.2%), and 6/9 (66.7%) participants failed both the pre-test and post-test timings for the triangle, cone, and ball tasks, respectively (Figure 6). There were 3/9 experimental participants (33.3%) who failed the cone tasks both times, and all 9 (100%) failed the ball tasks both times (Figure 6).

**Figure 7:**
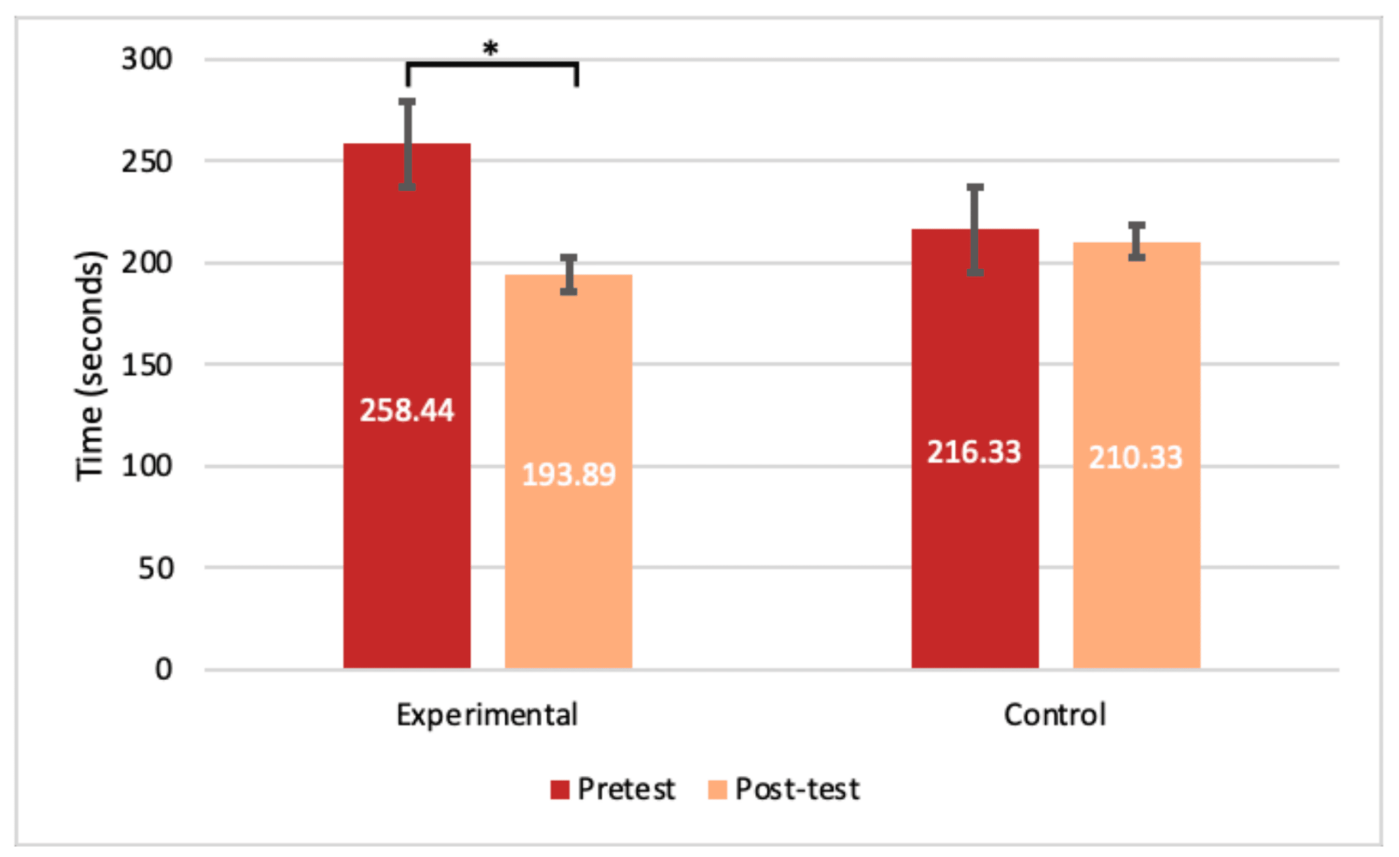
Average times for the triangle task. The average pre- and post-test timing results in seconds for the experimental and control groups for the triangle task. T-tests resulted in significance for the experimental group only (n=9) with a p<0.001. Error bars represent SEM. *significance with a p-value <0.05 SEM: standard error of the mean

**Figure 8:**
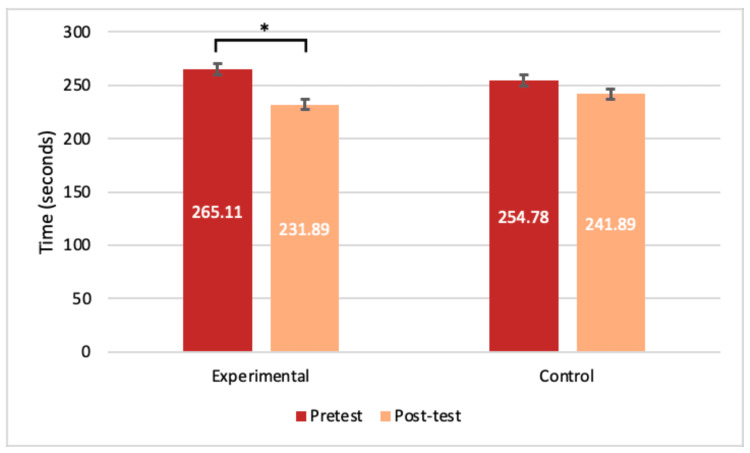
Average times for the cone task. Comparing the average pre- and post-test timing results in seconds for the experimental and control groups for the cones task. T-tests resulted in significance for the experimental group only (n=9) with a p<0.05. Error bars represent SEM. *significance with a p-value <0.05 SEM: standard error of the mean

Experimental participants had an average time of 258.44 seconds (SD= 52.131) on the pre-test triangle task; compared to the post-test re-timing, when they averaged 193.89 seconds (SD=54.33), a statistically significant decrease in time (p<0.001) (Figure 7). Control participants had an average time of 216.33 seconds (SD=67.24) for the triangle task pre-test and an average of 210.33 seconds (SD=72.65) for their post-test (Figure 7). For the cone task, experimental participants averaged 265.11 seconds (SD=60.84) for the pre-test trials and 231.89 seconds (SD=56.85) for the post-test trials, a statistically significant difference (p<0.05) (Figure 8). The control participants had no significant decrease in time between trials, averaging 254.78 seconds (SD=42.20) and 241.89 seconds (SD=62.58) for the pre-test and post-test cone tasks, respectively (Figure 8).

To assess whether the intervention resulted in greater improvement compared to the control group, independent t-tests were performed on the change in task completion time (pre-test to post-test) for each task. For the triangle task, the experimental group demonstrated a greater mean improvement compared to the control group (65.7 ± 39.9 seconds vs. 17.1 ± 66.2 seconds); however, this difference did not reach statistical significance (p=0.076). For the cone task, the experimental group also showed greater mean improvement compared to the control group (54.7 ± 63.0 seconds vs. 15.8 ± 78.5 seconds), though this difference was not statistically significant (p=0.283). For the ball task, no meaningful difference in improvement was observed between groups, as both groups demonstrated minimal change due to a floor effect (p=1.000).

Experimental participants reported using their nondominant hand the most for brushing their teeth. They reported the least amount of nondominant hand usage for writing on paper or a tablet. Using a light switch, eating finger foods, opening drawers, using doorknobs, picking up a cup, and brushing their hair had the highest number of reports of using both their dominant and nondominant hand equally throughout the day. Notably, unlocking doors with a key had the highest rate of responses for using the nondominant hand exclusively during the day. Additionally, 9/9 (100%) of experimental participants reported feeling substantially more confident in their laparoscopic abilities during the post-test.

## Discussion

This study explored the impact of nondominant hand training on laparoscopic task performance among preclinical osteopathic medical students. The findings suggest that intentional nondominant hand use in daily life over a three-week period may contribute to improvements in specific laparoscopic skills, particularly for tasks requiring bilateral coordination and precision. While both experimental and control groups showed some improvement in performance on simpler tasks like the triangle and cone placements, only the experimental group demonstrated a consistent increase in total task success from pre-test to post-test. Notably, the experimental group improved from 9 to 15 successful task completions. In contrast, the control group showed a decline in overall successful completions, despite initially performing better during the pre-test.

As expected, the triangle task, which was the simplest in terms of required fine motor skills, showed the highest improvement rates in both groups (Tables 1, 2). The pliability of the triangles makes them easily manipulated. However, the cone task, which requires more precise movements for the transfer and placement, demonstrated a greater relative improvement in the experimental group, with fewer participants slowing between pre- and post-test. These findings align with the hypothesis that practicing nondominant hand use may enhance bilateral motor coordination, which is essential in laparoscopic procedures. Interestingly, neither group showed any improvement on the ball task, which required the finest motor dexterity and control because of the intricate hand movements needed to maintain possession of the ball without squeezing too hard to lose control of it. This suggests that the level of complexity in that task may exceed the degree of ambidexterity achievable through short-term, low-intensity daily habit changes. All experimental participants failed this task both times, indicating that additional or more targeted training may be required to improve performance on high-dexterity tasks.

To compare the effect of the intervention between groups, independent t-tests were performed on change scores for each laparoscopic task. Although the experimental group demonstrated greater mean improvement than the control group across both the triangle and cone tasks, these differences did not reach statistical significance. This suggests that while a trend toward improved performance was observed following structured nondominant hand use, the effect was not strong enough to be statistically confirmed within the current sample size. The lack of statistical significance may be attributed to the small sample size and resulting limited statistical power, which may have reduced the ability to detect between-group differences. Additionally, variability in individual performance likely contributed to wide standard deviations, further influencing the ability to reach significance. Notably, no meaningful between-group differences were observed for the ball task, which exhibited a clear floor effect in both groups. This suggests that the level of task difficulty exceeded the skill gains achievable through short-term, unstructured nondominant hand training. Despite the absence of statistically significant differences, the consistent direction of improvement across simpler tasks suggests a potential beneficial effect of nondominant hand training on basic laparoscopic skill acquisition. These findings support the need for larger, adequately powered studies to further evaluate this intervention and determine its impact on more complex motor tasks.

Survey data supported participant engagement with the intervention. Most experimental group members reported frequently using their nondominant hand for tasks like brushing teeth and unlocking doors, which may involve consistent and intentional motor control. However, tasks like writing were avoided, likely due to the inherent difficulty and discomfort, suggesting a natural ceiling to self-directed hand training without formal instruction or structured practice. The variation in usage of experimental participants’ nondominant hands throughout the day could also have been influenced by using the string and the convenience of the task. Additionally, participants verbally acknowledged that they noticed they were using their nondominant hands for daily tasks even after the study concluded. Furthermore, all participants in the experimental group reported a subjective increase in confidence with laparoscopic tools after the post-test, despite minimal objective improvement on the most difficult task. This finding highlights the potential motivational or psychological benefits of incorporating ambidexterity-focused exercises into early surgical education, even when technical improvements are still developing. To ensure compliance beyond self-reported surveys, participants could be asked to maintain a daily activity log verified periodically by researchers. Additionally, incorporating wearable activity trackers or observational check-ins could provide more objective data on nondominant hand usage throughout the study period.

These findings are consistent with prior literature demonstrating that while nondominant hand training and ambidexterity-focused exercises may improve basic laparoscopic skill performance, the magnitude of improvement is often task-dependent. Studies in surgical simulation training have shown reductions in performance asymmetry between dominant and nondominant hands following structured practice, particularly for simpler psychomotor tasks, but more complex dexterity-based activities demonstrate limited or inconsistent improvement [6]. Similarly, a study by Saleh et al. highlights persistent performance differences between dominant and nondominant hands in laparoscopic simulation, suggesting that short-term or unstructured training may primarily influence foundational coordination rather than advanced bimanual proficiency [11]. Together, these findings support the present study’s observation that nondominant hand training may enhance basic task performance, but is insufficient on its own to improve high-dexterity laparoscopic skills.

There are several limitations to this study. The small sample size (n=18) limits the generalizability of the findings, and the participants' baseline skill levels were relatively uniform due to their preclinical status. Additionally, the reliance on self-reported compliance with the nondominant hand intervention introduces potential bias and variability in training intensity. The lack of improvement on the ball task suggests that three weeks of unstructured hand use may not be sufficient for developing the fine motor precision required for more complex laparoscopic tasks. Another limitation is the lack of a structured skills curriculum or reinforcement for nondominant hand use; participants were not supervised or coached in technique, which might have enhanced the training effect. Furthermore, due to the novelty of the tasks and tools, some improvement could have stemmed from general familiarity with the simulator itself rather than from true skill acquisition.

## Conclusions

This study suggests that short-term nondominant hand training through daily activities may support improvements in basic laparoscopic task performance and learner confidence among preclinical medical students. However, the absence of improvement in more complex tasks indicates that this approach alone may be insufficient for developing higher-level fine motor skills. These findings should be interpreted cautiously, as observed improvements may partially reflect increased familiarity with simulation tasks rather than true skill acquisition. Within these limitations, nondominant hand training may serve as a low-cost, accessible adjunct to early surgical education. Future studies should evaluate longer training durations, incorporate structured task-specific exercises, and assess effects on complex skill development and long-term retention across more diverse trainee populations.

## References

[1] Gilbert AN, Wysocki CJ (1992). Hand preference and age in the United States. Neuropsychologia.

[2] Peters M, Reimers S, Manning JT (2006). Hand preference for writing and associations with selected demographic and behavioral variables in 255,100 subjects: the BBC internet study. Brain Cogn.

[3] Boggio PS, Castro LO, Savagim EA (2006). Enhancement of non-dominant hand motor function by anodal transcranial direct current stimulation. Neurosci Lett.

[4] Philip BA, Thompson MR, Baune NA, Hyde M, Mackinnon SE (2022). Failure to compensate: patients with nerve injury use their injured dominant hand, even when their nondominant is more dexterous. Arch Phys Med Rehabil.

[5] Sobinov AR, Bensmaia SJ (2021). The neural mechanisms of manual dexterity. Nat Rev Neurosci.

[6] Nagaraj MB, AbdelFattah KR, Farr DE (2022). Laparoscopic ambidexterity in left-handed trainees. J Surg Res.

[7] Borod JC, Caron HS, Koff E (1984). Left-handers and right-handers compared on performance and preference measures of lateral dominance. Br J Psychol.

[8] Brunt LM, Warner BW (2016). Commentary on hand laterality and acquired ambidexterity in surgical training. Ann Surg.

[9] Muratori LM, Lamberg EM, Quinn L, Duff SV (2013). Applying principles of motor learning and control to upper extremity rehabilitation. J Hand Ther.

[10] Chung AT, Lenci LT, Wang K, Collins TE, Griess MD, Oetting TA, Shriver EM (2017). Effect of fine-motor-skill activities on surgical simulator performance. J Cataract Refract Surg.

[11] Saleh S, Uppal S, Sharma V (2015). Greater nondominant hand proficiency is not associated with enhanced simulated surgical performance. Can J Ophthalmol.

[12] Uswatte G, Taub E, Morris D, Vignolo M, McCulloch K (2005). Reliability and validity of the upper-extremity Motor Activity Log-14 for measuring real-world arm use. Stroke.

